# Applications of Artificial Intelligence in Myopia: Current and Future Directions

**DOI:** 10.3389/fmed.2022.840498

**Published:** 2022-03-11

**Authors:** Chenchen Zhang, Jing Zhao, Zhe Zhu, Yanxia Li, Ke Li, Yuanping Wang, Yajuan Zheng

**Affiliations:** Department of Ophthalmology, The Second Hospital of Jilin University, Changchun, China

**Keywords:** artificial intelligence, machine learning, deep learning, telemedicine, myopia

## Abstract

With the continuous development of computer technology, big data acquisition and imaging methods, the application of artificial intelligence (AI) in medical fields is expanding. The use of machine learning and deep learning in the diagnosis and treatment of ophthalmic diseases is becoming more widespread. As one of the main causes of visual impairment, myopia has a high global prevalence. Early screening or diagnosis of myopia, combined with other effective therapeutic interventions, is very important to maintain a patient's visual function and quality of life. Through the training of fundus photography, optical coherence tomography, and slit lamp images and through platforms provided by telemedicine, AI shows great application potential in the detection, diagnosis, progression prediction and treatment of myopia. In addition, AI models and wearable devices based on other forms of data also perform well in the behavioral intervention of myopia patients. Admittedly, there are still some challenges in the practical application of AI in myopia, such as the standardization of datasets; acceptance attitudes of users; and ethical, legal and regulatory issues. This paper reviews the clinical application status, potential challenges and future directions of AI in myopia and proposes that the establishment of an AI-integrated telemedicine platform will be a new direction for myopia management in the post-COVID-19 period.

## Introduction

With the continuous development of computer technology, big data acquisition and imaging methods, the application of artificial intelligence (AI) in medical fields is expanding. Recently, a large number of AI-related studies have been carried out in many disciplines, such as ophthalmology, radiology, cardiovascularology, and oncology (1–4). Thanks to the development of multimodal imaging, fundus photography and optical coherence tomography (OCT) have provided rich datasets for the development of AI models and have made it possible for AI to flourish in the field of ophthalmology. The study of diseases has expanded from initial diabetic retinopathy (5–8), age-related macular degeneration (9–11), and glaucoma (12–15) to anterior segment diseases, such as refractive error (16–18).

Refractive error, represented by myopia, is becoming a key public health issue. As any degree of myopia will increase the risk of adverse changes in eye tissue, high myopia and pathological myopia (PM) significantly increase the risk of irreversible visual impairment [e.g., glaucoma, retinal detachment, myopic macular degeneration (MMD), and macular choroidal neovascularization] or blindness (19). Early identification of high-risk groups of myopia and regular and repeated follow-up to document the progression of myopia and complications are essential for eye care providers to plan interventions. However, current healthcare systems may not be able to cope with the growing burden. In particular, the COVID-19 pandemic demonstrates the need for remote testing and monitoring. Fortunately, AI technology combined with telemedicine can bridge this gap. To date, studies have integrated AI into all stages of clinical practice of myopia and have achieved positive application effects. This paper introduces the concepts of AI, summarizes the clinical application status, discusses potential challenges and future directions of AI in myopia, and proposes that the establishment of an AI-integrated telemedicine platform will be a new direction of myopia healthcare to provide personalized management throughout the whole process for myopia patients in the post-COVID-19 period.

## AI, Machine Learning, and Deep Learning

The concept of AI was first proposed by John McCarthy in 1956. Its definition simulates human intelligence through machines (20). Machine learning (ML) is a branch of AI and mainly uses computer system programming to perform tasks or predict results (21). ML has great potential in clinical practice and machine translation (22). Traditional ML algorithms use variables selected by experts as input and usually do not involve large neural networks. They include algorithms such as linear regression, logistic regression, support vector machine, decision tree, and random forest algorithms (23). Deep learning (DL) is a subset of ML. Without special programming, it can automatically extract the rules from known data for the judgment of unknown data; hence, DL can process more complex data (24). DL algorithms usually involve the use of large-scale neural networks, such as artificial neural networks (ANNs), convolutional neural networks (CNNs) and recurrent neural networks (RNNs) (23). Since 2012, the introduction of CNNs has allowed for major breakthroughs in DL in imaging-based applications (e.g., object recognition, image segmentation, and disease classification) (24). VGG, ResNet, Inception and Inception-ResNet are some of the popular CNNs used for classification and are now widely used in medical image recognition (23). Deep CNNs can learn the feature representation from data without human knowledge and have the power to process large training data with high dimensionality. Studies have shown that the accuracy of medical image analysis systems based on DL in disease detection is equal to or even better than that of clinicians or trained personnel (25, 26). Moreover, other studies have proven the potential and feasibility of applying DL algorithms to disease screening and detection (27, 28). The diagnosis of many ophthalmic diseases requires not only symptom evaluation but also imaging information. This feature leads to the widespread use of AI technology represented by DL in clinical ophthalmology (1).

**Figure 1:**
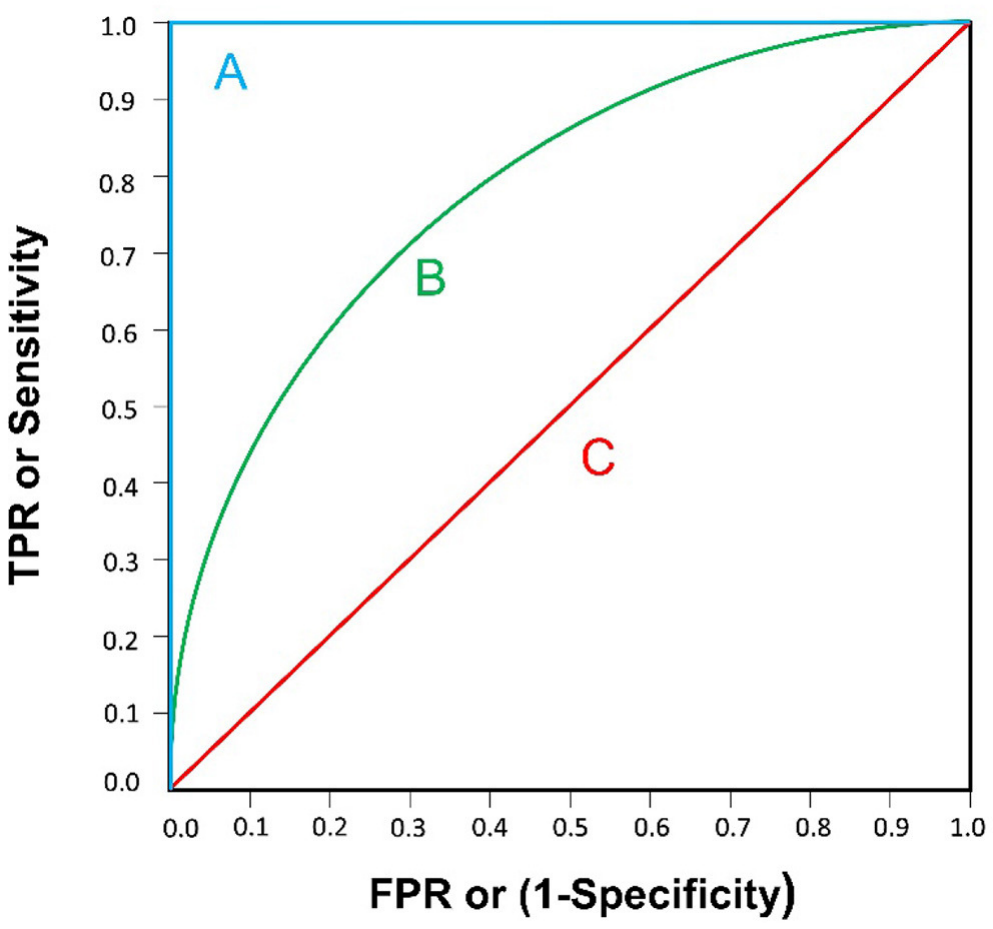
Three examples of ROC curve are illustrated. **(A)** AUC=1: A “perfect” classifier; **(B)** 0.5<AUC <1: A real-world classifier, better than random guess; **(C)** AUC=0.5: Like random guess (e.g., coin tossing), models have no predictive value.

The indexes used to evaluate the quality of an AI model are accuracy, sensitivity and specificity, which are calculated by using four quantitative indexes: true positive, false positive, true negative and false negative (Table 1). A receiver operating characteristic curve (ROC) can be drawn with the false positive rate (FPR) as the X-axis and the true positive rate (TPR) as the Y-axis. The area under the curve (AUC) is defined as the area under the ROC curve and generally ranges from 0.5 (for a model with no predictive value) to 1 (for a perfect model) (29) (Figure 1).

**Table 1:** Common terminologies used to evaluate AI model performance.

		**Predicted outcome**	
		**Disease**	**No disease**
**Actual outcome**	**Disease**	True positive (TP)	False negative (FN)
	**No disease**	False positive (FP)	True negative (TN)
**Remark**	Accuracy = (TP+TN)/(TP+FN+FP+TN)		
	Sensitivity = TP/(TP+TN)		
	Specificity = TN/(TN+FP)		
	True positive rate (TPR) = Sensitivity		
	False positive rate (FPR) = 1-Specificity		

## Global Burden of Myopia

Myopia is one of the most common ophthalmic diseases in the world. It mainly occurs in childhood and early adulthood (30). According to the work of Holden and his coworkers, the global prevalence of myopia is close to 28.3% (2 billion) of the world's population, of which 4.0% (277 million) suffer from high myopia. The “myopia epidemic” is estimated to affect 49.8% (4.758 billion) of the world's population by 2050, with 9.8% (938 million) suffering from high myopia (≤ -5.00 D). Of note, Holden et al. standardized to a spherical equivalent of 5.00 D or less for high myopia because it is widely used to identify people at higher risk of pathologic myopia (31). Nature (genetics and heredity) and nurture (environment and lifestyle) are all factors leading to myopia (19). For most people with myopia, the most critical risk factor is likely to be related to modern lifestyles, which include long periods of close-eye activity. The outbreak of COVID-19 at the end of 2019 undoubtedly exacerbated the above phenomenon. Research shows that during the COVID-19 pandemic, the reduced time spent outdoors and increased exposure to electronic screens have led to a further increase in the risk of myopia in children (32, 33).

Most cases of myopia are associated with excessive axial growth (19). Retinal damage caused by excessive axial growth is irreversible. Irreversible visual impairments caused by myopia (e.g., glaucoma, retinal detachment, MMD and macular choroidal neovascularization) or blindness not only increase medical costs but also reduce the quality of life of patients, which has caused a global medical and economic burden. Therefore, it is of great significance to comprehensively carry out myopia healthcare services, including the detection, diagnosis, progression prediction and treatment of myopia, as well as the management and prevention of ocular complications and visual impairment in patients with high myopia.

## AI in the Detection and Diagnosis of Myopia

### Refractive Error Assessment

To evaluate refractive error, traditional visual acuity examinations are not only time consuming and laborious but also rely on expensive machines and experienced doctors and technicians. People with expression difficulties (e.g., young children, the elderly, and patients with verbal communication disabilities) have particular difficulties cooperating during an examination (34). In developing countries or impoverished areas, the lack of doctors and medical equipment makes it difficult to accurately evaluate refractive error, and patients are likely to miss the optimal treatment window, resulting in an irreversible loss of vision. Thus, providing timely and high-quality refraction services that are accepted by the general population is extremely needed.

While it is generally difficult for ophthalmologists to evaluate refractive error from a retinal fundus photograph, DL techniques are capable of predicting them fairly accurately. Varadarajan et al. (16) trained a DL algorithm to predict refractive error from retinal fundus photographs. By analyzing attention maps to determine the parts of a photograph most relevant for prediction, they concluded that attention maps consistently highlighted the fovea as a feature that was important for prediction. Tan et al. (35) also reported that by using color fundus photographs, a system consisting of a CNN pretrained with the XGBoost algorithm was able to evaluate refractive error with a high degree of accuracy. Yang et al. (17) trained a DL system to detect myopia automatically from ocular appearance images, and the system obtained an AUC of 0.9270. The research demonstrated the possibility of screening and monitoring refractive status in children with myopia in remote areas.

### The Diagnosis of Pathologic Myopia and Complications

PM is accompanied by degenerative changes in the retina, which, if left untreated, can lead to irrecoverable vision loss. It is essential for ophthalmologists to have a sustainable method of monitoring eyes with PM to reduce blinding complications, especially given that many PM patients are young or middle aged. However, the diagnosis of PM, defined as peripapillary atrophy and myopic maculopathy, generally requires a complete examination that includes an assessment of the visual acuity and color fundus photograph acquisition tasks that are labor intensive and skill-dependent (36).

Tan et al. (37) introduced a method to automatically detect PM *via* peripapillary atrophy features by means of variational level sets from fundus photographs. To improve prediction accuracy, Zhang et al. (38) proposed a computer-aided framework based on an ML algorithm for the detection of PM. By analyzing demographic and clinical information, retinal fundus photograph data and genotyping data from 2,258 subjects, this method achieved an AUC of 0.888 and outperformed the detection results obtained from the use of demographic and clinical information (an AUC of 0.607), imaging data (an AUC of 0.852) or genotyping data (an AUC of 0.774) alone, with increases of 46.3%, *p* < 0.005; 4.2%, *p* = 0.19; and 14.7%, *P* < 0.005, respectively. Recently, Hemelings et al. (39) developed a successful approach based on a DL algorithm for the simultaneous detection of PM, with an AUC of 0.9867, and the segmentation of myopia-induced lesions. Other similar studies have also been reported, such as those identifying the different types of lesions of myopic maculopathy automatically from fundus photographs with DL models (40, 41). In addition, OCT macular images were used for the development of CNN models to identify vision-threatening conditions, such as retinoschisis, macular holes and retinal detachment, in adults with high myopia, and the models obtained good sensitivity and AUC scores (42, 43).

## AI in the Prediction of Myopia Progression

Considering the potential irreversible disease burden during adulthood, concerns from parents, clinicians and policy makers include the potential progression rate and risk of developing high or even pathological myopia from childhood myopia (44). Thus, predicting myopia progression can provide evidence for transforming clinical practice, health policy-making, and precise individualized interventions regarding the practical control of school-aged myopia.

Lin et al. (45) identified myopia development rules and predicted the onset of myopia and its progression for children and teenagers from clinical measures using a random forest ML model, which had good predictive performance (the AUC ranged from 0.801 to 0.837) for up to 8 years in the future. Yang et al. (46) developed a prediction model to predict myopia in adolescents based on both measurement and behavior data of primary school students, and the model achieved reasonable performance and accuracy. Further research is still required for interpopulation validation to allow these models to be generalized.

## AI in Refractive Surgery for Myopia

The aim of refractive surgery is to correct refractive error in adults with stable myopia and reduce their dependence on corrective aids. Keratorefractive procedures and intraocular procedures are two main forms of refractive surgery. At present, keratorefractive procedures include laser epithelial keratomileusis (LASEK), laser *in situ* keratomileusis (LASIK) and small incision lenticular extraction (SMILE). Intraocular procedures include phakic intraocular lens (PIOL) implantation and cataract surgery (19). To achieve the goal of optimal visual and refractive outcomes and to minimize the risk of postoperative complications, researchers have creatively applied AI to various stages of refractive surgery and achieved ideal results, particularly in the preoperative screening for risk of ectasia following LASIK, guiding the formulation of surgical plans and intraocular lens (IOL) power calculations.

### Preoperative Screening

In 1998, Seiler et al. (47) published the first reports of iatrogenic progressive ectasia after LASIK, also known as iatrogenic ectasia. This complication can cause postoperative refraction regression and seriously affect the operation effect. Ectasia occurs due to biomechanical decompensation of the stroma, which may be related to pre-existing biomechanical weakening (e.g., keratoconus, subclinical keratoconus, and forme fruste keratoconus) or a severe impact on the corneal structure (e.g., an attempted treatment for high myopia) (48). Screening before refractive surgeries is extremely important to identify candidates at high risk of iatrogenic ectasia. Xie et al. (49) combined a DL algorithm with corneal tomographic scans to develop the Pentacam InceptionResNetV2 Screening System to screen potential candidates for refractive surgery. They reported a sensitivity of 80% for identifying ectasia suspects, 90% for diagnosing early keratoconus, and an overall diagnostic accuracy of 95% with an AUC of 0.99. To train and develop more accurate AI-based algorithms for identifying candidates at high risk of iatrogenic ectasia, it is necessary to have a longitudinal follow-up and collect more clinical data to train and validate the AI models.

### Guiding the Formulation of a Surgical Plan

AI technology can guide a surgeon in selecting the best corneal refractive surgery method to perform on a specific patient. Yoo et al. (50) developed an expert-level multiclass ML model for selecting refractive surgery options for patients. They classified patients into LASEK, LASIK, SMILE and contraindication groups. Using data from 18,480 subjects who intended to undergo refractive surgery, the model was trained to select the optimal refractive surgery type for patients with accuracies of 81 and 78.9% on the internal and external validation datasets, respectively. Cui et al. (51) developed an ML model to recommend a nomogram for SMILE surgery to achieve the optimal postoperative visual outcome. They reported that the efficacy index in the ML group (1.48 ± 1.08) was significantly higher than that in the surgeon group (1.3 ± 0.27) (*t* = −2.17, *P* < 0.05). For high myopia patients who intend to undergo PIOL surgery, which involves the insertion of an additional lens in the anterior segment, it is essential to have correct anterior chamber depth (ACD) measurement (52). ACD measurement is usually obtained with conventional A-scan ultrasound. However, these machines are expensive and cumbersome and may not be available in remote areas. Chen et al. (53) developed a new method for predicting central ACD using a portable smartphone slit lamp device aided by ML. This novel device may provide a new perspective to increase the convenience of ACD measurement.

### IOL Power Calculation Related to Myopia

For patients who intend to undergo PIOL implantation or cataract surgery to correct refractive error, accurate IOL power is the key to improving their postoperative visual quality. Ongoing developments in IOL power calculation incorporate new technology and data science to improve the accuracy of IOL selection (54). Compared with the second- and third-generation formulas, fourth-generation formulas, such as the Olsen formula (based on ray tracing) and Barrett Universal II (BUII), show good accuracy and fewer refractive accidents (55). A recent study developed a new XGBoost ML-based calculator for highly myopic eyes, which incorporated the BUII formula results and showed a significant improvement in the percentage of eyes achieving ±0.25 D of the prediction error compared with the BUII formula alone (18). To date, for high axial myopia, AI-based IOL formulas seem to demonstrate higher levels of accuracy, including the Hill-radial basis function (RBF) calculator and the Kane formula (56–59). The Hill-RBF calculator uses AI and regression analysis with a very large database of actual postsurgical refractive outcomes to predict IOL power (59). Hill-RBF is based mainly on empirical data; thus, its accuracy is limited by the type of data and eye characteristics from which it is derived (54). To overcome this limitation, Hill-RBF 2.0 expanded the database and improved IOL power prediction for a wider range of eye characteristics, such as high axial myopia, by continuously collecting various eye characteristics and surgical results (57). In September 2020, Hill-RBF 3.0 was released. With the expansion of the Hill-RBF database, the calculator is more likely to obtain a better accuracy in IOL power prediction. The other promising method for IOL calculation is the Kane formula, which incorporates AI with theoretical optics to predict IOL power (54). Studies have shown that the Kane formula has a smaller absolute error than the BUII, Olsen, and Hill-RBF 2.0 formulas (60, 61). In a study of 10,930 eyes in Britain, the Kane formula had the lowest mean absolute prediction error for all ranges of ALs and obtained the smallest absolute error for long eyes (AL>26.0 mm) (60).

## AI and Monitoring Devices in the Behavioral Intervention of Myopia

Effective behavioral intervention is as important as early detection to prevent myopia or limit myopia progression. To understand behaviors related to myopic onset and progression, a wearable device named Vivior Monitor (Vivior AG, Zurich, Switzerland) was developed to investigate the visual behavior of children with myopia (6–16 years old) (62). Using ML algorithms, Vivior Monitor identified types of visual activities, such as viewing handheld media, desktop work, and computer work. This research reported that older children spent less time viewing objects at distances, more time using a computer and less time engaging in physical movement. There is no doubt that outdoor activity is the main protective factor against myopia (63, 64). Wearable devices in combination with internet or social network apps aimed at encouraging children to spend more time outdoors are now being developed. The Singapore Eye Research Institute developed a novel wearable fitness tracker (FitSight), which comprises a smartwatch (Sony Smartwatch 3; Sony Corp., Minato, Tokyo, Japan) with a light sensor and an accompanying smartphone app that logs time spent outdoors and sends feedback to parents and children (65). In addition, excessive near-work behavior is one of the most commonly known unhealthy visual behaviors related to myopia, and many studies have shown that it can speed the occurrence and development of myopia (66, 67). Clouclip (Glasson Technology Co. Ltd., Hangzhou, China), a cloud-based sensor device that attaches to the sides of spectacles, can objectively and dynamically monitor the wearer's near-work distance and duration (68, 69). This device can provide a vibration alert when it detects risky near-work-related behaviors, such as particularly short viewing distances or prolonged continuous near-work behavior. Cao et al. (68) collected data from 67 subjects who were assigned to wear Clouclip all day (except for bathing and sleeping) during the experiment; they found that the device can significantly modify near-work behaviors in school-age children and that its effects can last a certain period of time.

## AI in Myopia Genetics

The mechanism of myopia is extremely complex. Nature (genetics and heredity) and nurture (environment and lifestyle) are all factors leading to myopia (19). In recent years, studies on the genetics of myopia have also received considerable attention. By linkage analysis, candidate gene analysis, genome-wide association study (GWAS) and next-generation sequencing (NGS), more than 100 genes and over 20 chromosomal loci have been identified to be associated with myopia or related quantitative traits (70–72). However, the current knowledge about the genetic contributions of the loci and genes to myopia remains limited (73).

To date, studies using big data for genetic analysis and phenotyping correlation have achieved significant progress in various medical fields (74, 75). Genomic readouts, combined with advanced AI, could be a powerful approach for risk prediction in multifactorial diseases such as myopia. At present, both CNNs and RNNs have shown considerable potential in a variety of clinical genomics applications, such as variant calling, genome annotation, and functional impact prediction (76). Given the diversity of myopia with regard to its environmental burden, geographic patterns, and affiliations with different ethnicities and cultural groups worldwide (73), further AI research with larger multiethnic genetic samples from various research institutes will be essential to drive the discovery of new insights into the genetic aspects of myopia and advance AI-genomic applications in managing childhood myopia (77).

## New Model For Myopia Management: Telemedicine

Telemedicine is a new service model in the medical field that aims to solve the problem of healthcare for people in remote and underdeveloped areas by providing remote medical services (78). The global COVID-19 pandemic is bringing telemedicine to the forefront of ophthalmic medical services (79, 80). With the development of AI technology and the expansion of 5G communication network coverage, AI-integrated telemedicine platforms will gradually become the new normal of post-COVID-19 ophthalmic care. In the clinical application of myopia, AI-integrated telemedicine platforms should mainly focus on the following aspects: reducing the manpower requirements of ophthalmic clinics, reducing the risk of direct physical contact between patients and doctors, and personalizing management throughout the whole process.

Devices based on AI enable non-ophthalmologist health care workers, such as optometrists, nurses and technicians, to perform several tasks, such as assessments of refractive error and measurements of ACDs, individually instead of patients moving through a number of different clinical staff, each performing a specific task. In addition, telemedicine can not only reduce the direct physical contact between patients and doctors but also prolong the distance of ophthalmic examination. For example, the portable slit lamp examination distance has increased from 18 cm to 55 cm. The examination distance increased from 5 cm for the direct ophthalmoscope to 47 cm for the Glasgow Retinal Imaging Adaptor (Medical Devices Unit, NHS Greater Glasgow & Clyde, UK) (81). These changes can not only satisfy the need for regular and repeated follow-up to monitor and document the refractive status of myopia with high efficiency but also limit exposure risks.

**Figure 2:**
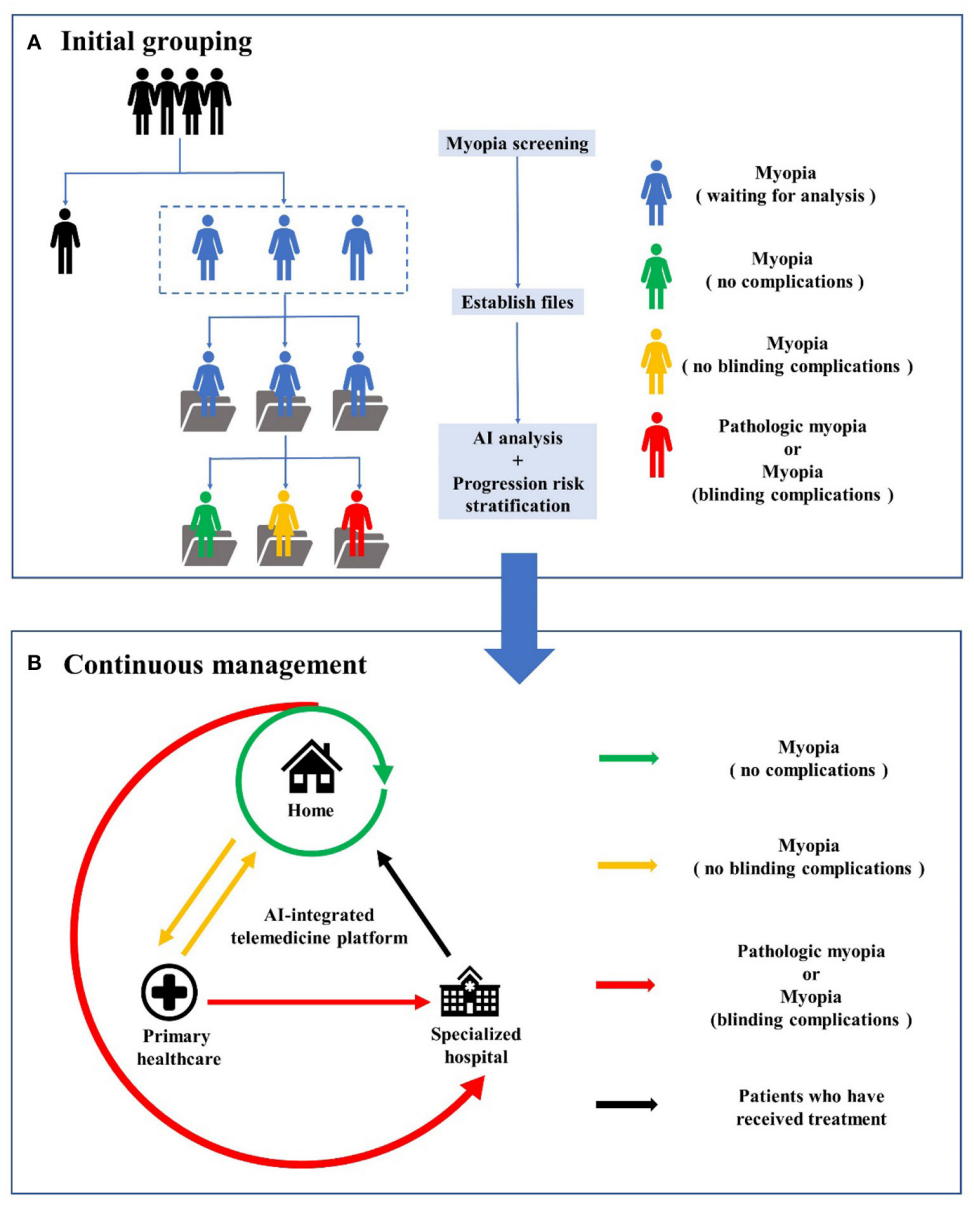
The workflow of AI-integrated telemedicine platform for myopia. **(A)** is the workflow of initial grouping, including myopia screening, files establishing, AI analysis and progression risk stratification for myopia patients. **(B)** is the workflow of continuous management involving self-monitoring at home, primary healthcare and specialized hospital services.

To provide personalized management for myopia patients throughout the whole process, we first need to realize the integration of hospital-community-family health management. Recently, Wu et al. (82) proposed an AI-integrated telemedicine platform to screen and refer patients with cataracts. According to the authors, this telemedicine platform involves self-monitoring at home, primary healthcare and specialized hospital services. Inspired by this platform, we propose a new management model for myopia (Figure 2). First, considering that myopia develops primarily during childhood and early adulthood, large-scale refractive error screening of the target population will be carried out regularly with portable devices and technologies based on AI, and the examination data will be stored and documented on telemedicine platforms. Second, AI analysis will be conducted on the collected clinical data, images and potential genomic data to classify the risk of myopia progression in clinically identified individuals and formulate personalized management plans, including visual behavioral interventions for patients with wearable devices (77). Third, home monitoring (using ocular appearance images taken by family members with cell phones and visual acuity tests) can be implemented for patients without myopia-related complications. Home monitoring and community-based primary healthcare institutions (where retinal fundus photographs or OCT scans are captured and used in the telemedicine platform with AI analysis) can be used by myopia patients with non-blinding myopia complications. If the above patients develop pathological myopia or myopia with blinding complications, they can be referred to the specialized hospital *via* a fast tract notification system. Patients initially diagnosed with pathological myopia or blinding complications should be directly transferred to tertiary medical institutions. After treatment, the patient returned home and continued home monitoring. Fourth, for patients requiring surgical treatment, AI-integrated telemedicine can be applied to preoperative screening to determine the risk of ectasia following LASIK and guide the formulation of a surgical plan and IOL power calculation.

## Current Challenges and Future Directions

Despite the reported successful clinical applications of AI in myopia, challenges and hurdles are still present. Critical technical and clinical limitations must be surmounted prior to the widespread implementation of AI in myopia.

### Standardization of Datasets

Image-based AI technology has made some progress in the application of refractive error assessment, screening, diagnosis and treatment of myopia. However, image-based AI requires large, standardized, labeled data, and ophthalmic open datasets are very small compared to ImageNet's tens of millions of images (13). Obtaining large-scale and high-quality images in a real clinical environment is a great challenge. Technically, more advanced data enhancement methods should be utilized, such as programming simulated lesions to be integrated into normal image data (83) or incorporating real lesions into other locations in normal or abnormal images (84). Recent studies have proposed alternative training methods that can learn from less data. For example, some studies synthesize a large number of random and diverse medical images by generative adversarial networks and report that these images can be used as CNN training datasets in the future (85–87). However, these new methods have not achieved significant success thus far, and their effectiveness needs to be further proven (88). In addition to the amount of data, the quality of images also plays a great part in the performance of AI models (5, 89). Research has reported that poor-quality fundus images that were not removed from the dataset were found to decrease the AUC by 0.1 (90). To surmount this challenge, Wu et al. (23) proposed a quality assessment system for images to select high-quality images. The feasibility of this method needs further study.

### Attitude Toward AI

As DL is an end-to-end learning method, that is, inputting original data and outputting results directly without manual coding, DL lacks the ability to explain the detection results and cannot provide an exact judgment basis for the results; this is called the “black box phenomenon.” This could reduce the acceptance of test results by ophthalmologists and patients (91). With the development of DL, several approaches are currently available to help improve the interpretability of the results, including occlusion tests (92) and saliency maps (93). However, there is no consensus on which saliency map generation method is most appropriate for ophthalmic imaging data (93). In addition, it is unclear how one should interpret non-traditional features identified by saliency analysis, that is, whether they should be treated as novel biomarkers or erroneous correlations “learned” during training. Processes need to be in place to address such disagreements, such as an independent third party from a multidisciplinary team, as would occur where there is clinical uncertainty (36). Apart from that, education on the implementation and appraisal of AI systems should be included in medical school programs and hospital training to prepare for its adoption when the technology reaches maturation for ophthalmology clinical practice.

### Ethical, Legal, and Regulatory Issues

With the increasing use of AI, security and privacy have become issues of concern and involve ethical, legal and regulatory issues (94). For example, an AI algorithm, similar to a human ophthalmologist, is definitely prone to errors. Who is responsible for bearing the legal consequences of an undesirable outcome due to an erroneous judgment made by an AI algorithm? Is it the company that develops the algorithm, the individual physician who utilizes the algorithm, or the healthcare organization under which the physician is employed (95)? In addition, protocols and laws aimed at guaranteeing training data and testing data security in AI need to be continually established and improved.

## Conclusions

Given the rapid increases in the prevalence of all levels of myopia in the past three decades and the non-linear rapid COVID-19 disease expansion, there is a need to revolutionize healthcare systems worldwide. Three main areas are the targets for such revolutions: improving efficiency, limiting exposure risk, and providing individualized management for myopic patients. AI is among the most promising solutions to address these issues. Prior to the mass adoption of AI in myopia, AI models need to be further optimized to improve their interpretability, human–machine interactions, generalization abilities, and robustness. It is also necessary to develop relevant clinical standards, integrate large-scale clinical datasets, and develop a standard evaluation framework for AI models in clinical practice. Moreover, relevant laws and regulations need to be constantly improved to achieve comprehensive supervision of practical applications.

## Author Contributions

CZ, JZ, and ZZ conceived the idea for the article and contributed to the initial drafting of the manuscript. YL, KL, and YW performed literature search and collected data. YZ was involved in reviewing and editing the manuscript. All authors read and approved the final manuscript.

## Funding

This work was supported by the Science and Technology Development Plan Project of Jilin Province (No. 20190303186SF).

## Conflict of Interest

The authors declare that the research was conducted in the absence of any commercial or financial relationships that could be construed as a potential conflict of interest.

## Publisher's Note

All claims expressed in this article are solely those of the authors and do not necessarily represent those of their affiliated organizations, or those of the publisher, the editors and the reviewers. Any product that may be evaluated in this article, or claim that may be made by its manufacturer, is not guaranteed or endorsed by the publisher.

## Abbreviations

AI: Artificial intelligence
OCT: Optical coherence tomography
ML: Machine learning
DL: Deep learning
ANNs: Artificial neural networks
CNNs: Convolutional neural networks
RNNs: Recurrent neural networks
ROC: Receiver operating characteristic curve
FPR: False positive rate
TPR: True positive rate
AUC: Area under the curve
5G: 5th generation mobile communication technology
MMD: myopic macular degeneration
PM: Pathologic myopia
LASEK: Laser epithelial keratomileusis
LASIK: Laser *in situ* keratomileusis
SMILE: Small incision lenticular extraction
PIOL: Phakic intraocular lens
IOL: Intraocular lens
AL: Axial length
ACD: Anterior chamber depth
BUII: Barrett Universal II
RBF: Radial basis function.
